# Sensitivity of clinical isolates of Candida to essential oils from Burseraceae family

**DOI:** 10.17179/excli2014-621

**Published:** 2016-04-19

**Authors:** Miloš Nikolic, Marija Smiljkovic, Tatjana Markovic, Ana Cirica, Jasmina Glamoclija, Dejan Markovic, Marina Sokovic

**Affiliations:** 1 Institute for Biological Research "Siniša Stankovic", University of Belgrade, Bulevar Despota Stefana 142, 11000, Belgrade, Serbia; 2 Institute for Medicinal Plant Research "Josif Pancic", Tadeuša Košcuška 2, 11000 Belgrade, Serbia; 3 Faculty of Dental Medicine, Department of Pediatric and Preventive Dentistry, University of Belgrade, dr Subotica 8, 11000 Belgrade, Serbia

**Keywords:** Candida spp., susceptibility, essential oils, Burseraceae, oral candidosis

## Abstract

The aim of this study was to investigate the chemical composition and antifungal activity of four commercial essential oils from the *Burseraceae* family - two *Boswellia carterii *Flueck oils, *Canarium luzonicum* (Blume) A. Gray oil, and *Commiphora myrrha* (Nees) Engl oil, against most common *Candida* spp. recovered from the human oral cavity. The essential oil samples were analyzed by GC-FID and GC/MS. The analysis showed that major essential oils' components were α-pinene (23.04 % and 31.84 %), limonene (45.62 %) and curzerene (34.65 %), respectively. Minimum inhibitory (MIC) and minimum fungicidal (MFC) concentrations were determined using a microdilution standardized technique. All tested *Candida *spp. clinical isolates and ATCC strains showed susceptibility to tested essential oils in a dose dependent manner. The strongest antifungal activity was shown by essential oil of *B. carterii, *sample 2; the average MIC values ranged from 1.25 to 1.34 mg/ml, and MFC values ranged from 2.50 to 3.75 mg/ml, depending on the fungus. This study supports the possible use of essential oils from the *Bursecaceae *family in reduction and elimination of *Candida* spp. populations in patients with oral cavity fungal infections.

## Introduction

Among the hundreds of microbial species from the oral cavity, members of the genus *Candida* are representative of several yeast species considered to be commensal oral microbiota. Among them, *Candida albicans* is the most commonly isolated from the human oral cavity (Back-Brito et al., 2009[6]), while the non-*albicans* species, such as *C. glabrata*, *C. krusei*, *C. tropicalis *and *C. dubliniensis *are generally less frequent, although there are indications that the incidence of non-*albicans *species recovered from cases of oral candidosis is increasing (Samaranayake, 1991[32]; Scully et al., 1994[35]).

Under certain circumstances *Candida *spp. commensals become pathogens and cause mucous membrane infections ranging from pseudomembranous candidodis and denture-induced stomatitis (Lam et al., 2012[21]; Gonsalves et al., 2007[15]; Da Costa et al., 2006[12]; Ben-Aryeh et al., 1980[8]), to life-threatening systemic diseases (Samaranayake and Yaacob, 1990[33]), particularly in immunocompromised patients with AIDS, cancer and *diabetes mellitus *(Seneviratne et al., 2008[36]; Edmond et al., 1999[13]).

Oral candidosis is an opportunistic infection usually accompanied by various symptoms including burning, painful sensation, change of taste and swallowing difficulty, but it can be also asymptomatic. It could be treated with various synthetic antifungal agents, though they possess some disadvantages such as high toxicity to the host tissues, emergence of drug-resistant species, and high cost (Runyoro et al., 2006[29]). There is a number of case reports describing the colonization and infection of immunocompromised patients or denture wearers subjected to long-term regimens of oral antifungal agents, from which drug less-responsive or resistant (Sheehan et al., 1999[37]; Wingard et al., 1991[44]; Wingard, 1994[43] and 1995[42]; Webb et al., 1998[41]; Sanglard and Odds, 2002[34]; Sullivan et al., 2004[38]), and even cross-resistant *Candida* spp. (Cross et al., 2000[11]; Magill et al., 2006[23]) have been recovered. In addition, recurrences of the oral candidosis, which are commonly observed in these patients, make the problem even greater (Taplin, 1976[40]).

Limitations of synthetic antifungal drugs have encouraged development of new classes of antifungal medications based on potent bioactive molecules of natural origin with high therapeutic efficiency, low toxicity, wide spectrum of activity and eco-friendly nature (Saini et al., 2008[31]; Rajeshkumar and Sundararaman, 2012[28]). Since plant-derived essential oils have a long tradition of use and cover a broad spectrum of biological activities, in various studies their efficacy has been tested over a wide range of oral bacteria and fungi (Hammer et al., 1999[16]; Carvalhinho et al., 2012[9]). Their natural origin, low cytotoxity and biodegradability made them potential ingredients in novel antifungal medications intended for use in oral hygiene maintenance, and prevention and management of oral infections. 

In addition, mindful of increased resistance of *Candida* spp. to synthetic antifungal drugs (Pina-Vaz et al., 2004[27]; Zomorodian et al., 2011[45]), testing the susceptibilities of clinical isolates to natural products such as essential oils, has contributed to development of novel antifungal treatments intended for use at target sites. Our approach also supports the idea that apart from classical antifungal treatments, combination therapy and preventive therapy represent emerging strategies for treating invasive fungal infections (Rüping et al., 2008[30]).

The aim of this study was to investigate the chemical composition and antifungal activity of four commercial essential oils from the *Burseraceae* family against the most common *Candida* spp. recovered from the human oral cavity.

## Material and Methods

### Essential oils

Four commercial essential oils belonging to the *Burseraceae* family were used in this experiment: 1) *Boswellia carterii *Flueck., sample 1 (EOBC_1_), purchased from an herbal pharmacy in Rotterdam, Holland; 2) *Boswellia carterii *Flueck., sample 2 (EOBC_2_), purchased from Sensient Essential Oils Germany GmbH, Bremen, Germany; 3) *Canarium luzonicum *(Blume) A. Gray, (EOCL) also purchased from Sensient Essential Oils Germany GmbH; and 4) *Commiphora myrrha* (Nees) Engl. (EOCM), also purchased from Sensient Essential Oils Germany GmbH.

### Essential oil analyses procedure

The essential oil analyses procedure for Gas Chromatography coupled with a Flame-Ionization Detector and Gas Chromatography/Mass Spectrometry analyses meets standards ISO 7609:1985[20], ISO 11024-1:1998[18], and 11024-2:1998[19], and they have been previously reported by Nikolić et al. (2013[25]).

GC-FID analysis was carried out using a GC Agilent Technologies 7890A apparatus, equipped with the split-splitless injector and automatic liquid sampler (ALS), attached to HP-5 column (30 m x 0.32 mm, film thickness 0.25 µm) and fitted with a flame-ionization detector (FID). Operating conditions were: H_2_ was the carrier gas (1 ml/min/ 210 °C); injector and detector T were 250 °C and 280 °C, respectively, while the column T was linearly programmed 40-260 °C at 4 °C/min. Samples of essential oils were first dissolved in ethanol (approx. 1 %) and then injected by ALS (1 µl, split-mode). Presence of the oils' compounds were calculated from the peak areas attained in corresponding area-percent reports (results of the standard processing of chromatograms), without correction factors.

The GC/MS was carried out on an HP G1800C Series II GCD analytical system equipped with a column HP-5MS (30 m x 0.25 mm, film thickness 0.25 µm). He was the carrier gas (1 ml/min). Other chromatographic conditions were identical to those for GC-FID. The transfer line was heated at 260 °C, while the mass spectra were recorded in EI mode (70 eV), ranging from 40 to 450 m/z. Essential oil samples were dissolved in ethanol (approx. 1 %) and then injected by ALS (0.2 µl, split mode).

Identification of the oils' compounds was found on matching of their mass spectra peaks with those from libraries of the Wiley 275 and NIST/NBS. The experimental Kovats' retention indices (RI values) were obtained by the use of calibrated Automated Mass Spectral Deconvolution and Identification System software (AMDIS ver. 2.1, 1997[4]), and compared to those from corresponding literature (Adams, 2007[3]), as an additional tool to the MS findings.

### Microorganisms

Clinical isolates of *Candida *spp. (58) and reference strains, *Candida albicans* ATCC 10231 and *Candida tropicalis* ATCC 750 (from Laboratory for Mycology, Institute for Biological Research “S. Stanković”, University of Belgrade, Serbia), were used in this study. The clinical isolates were collected by rubbing sterile cotton swabs over the oral mucosa of randomly chosen patients at the Faculty of Dental Medicine, University of Belgrade, Serbia. For collecting clinical isolates, used swabs were transferred to SD broth medium which were then thoroughly mixed using a vortex mixer; 50 μl of suspensions were subsequently inoculated on various selective and non-selective media and incubated microaerobically for 48 h at 37 °C. The isolates were identified using biochemical profiles with API 20C (bioMérieux France) and Chrom-Agar (Liofilchem sr.l. Italy).

### Anticandidal activity

Minimum inhibitory concentrations (MIC) and minimum fungicidal concentrations (MFC) were established by the use of microdilution standardized technique, EUCAST (2002[14]) with modification. In brief, with the use of sterile saline, fresh overnight yeast cultures were adjusted to a concentration 1.0 x 10^5^ CFU/per well. The microplates were left at 37 °C for 24 h. Following the addition of 40 μl P**-**Iodonitrotetrazolium violet (INT) 0.2 mg/ml (Sigma I8377) and 30 min of incubation at 37 °C, the MIC of samples were determined. 

The MIC values were considered as the lowest concentrations without microscopically observed growth. Following the serial sub-cultivations of 10 µl into microtitre plates containing 100 µl of broth/well, as well as subsequent 24 h incubation at 37 °C, the lowest concentrations with no visible growth were defined as the MFC values, indicating 99.5 % killing of the original inoculum.

A mouth rinse with active ingredient chlorhexidine 0.05 % (Curasept ADS 205, Curaden International AG, Kriens, Swiss) was used as a positive control. It is recommended for prophylactic use in order to maintain good oral hygiene in seriously diseased patients (Langslet et al., 1974[22]).

## Results

### Essential oil composition

The results of GC/MS analysis of commercial essential oils' samples are presented in Table 1(Tab. 1).

The main EOBC_1_ components, (i.e. contributing > 5 %), proved to be α-pinene, followed by limonene > α-thujene > *p*-cymene > sabinene > *trans*-β-caryophyllene, all together comprising 78.5 % of the oil (73.2 % monoterpene and 5.4 % of sesquiterpene hydrocarbons). The main EOBC_2_ components were α-pinene, followed by caryophyllene oxide > limonene > *trans*-β-caryophyllene > δ-cadinene, which accounted for 59.2 % of the oil (33.9 % monoterpene hydrocarbons, 14.1 % sesquiterpene hydrocarbons and 11.1 % oxygenated sesquiterpenes). The most dominant components of EOCM, typical for this oil were curzerene, followed by furanoeudesma-1,3-diene > lindestrene, which together accounted for 77.6 % of the entire oil (all oxygenated furano-sesquiterpenes). The main component of EOCL was limonene, followed by elemol > elemecin > α-phellandrene, together comprising 79.2 % of the oil (51.6 % monoterpene hydrocarbons, 21.4 % oxygenated sesquiterpenes and 6.2 % phenylpropane).

### Susceptibility of Candida spp. to essential oils

In general, all clinical isolates and ATCC strains of *Candida* spp. proved to be susceptible to tested *Burseraceae* family essential oils in a dose dependent manner. According to the lowest calculated average MIC and MFC values (2.19 mg/ml and 4.38 mg/ml, respectively) it appears that *C. krusei*, ATCC750 and ATCC10231, were generally more, and all of them, evenly susceptible to application of *Burseraceae* oils, followed by generally more endurable to the oils *C. albicans *(MIC 2.31 mg/ml and MFC 4.68 mg/ ml) and *C. glabrata* (MIC 2.66 mg/ml and MFC 5.31 mg/ml). In comparison to the control treatment with chlorhexidine mouth rinse (MIC 1.02 mg/ml and MFC 2.04 mg/ ml, for all tested fungi), susceptibility of tested clinical isolates and ATTC strains to *Burseraceae* oils was generally lower. 

Similar trends of susceptibility of all tested fungi were observed between the oils, although specific oil efficacy varied depending on the tested fungus. Based on calculated average MIC and MFC values, the most efficient essential oil proved to be EOBC_2_ (MIC 1.39/MFC 2.79 mg/ml). The least susceptible species on application of EOBC_2_ was a clinical isolate of *C. glabrata *(average MIC 1.88/MFC 3.75 mg/ml), followed by a *C. albicans* isolate (average MIC 1.34/MFC 2.68 mg/ml). The remaining three fungi, *C. krusei*, ATCC750 and ATCC10231, again expressed the same susceptibility trend, also proved by their equally expressed average MIC and MFC values (1.25 and 2.50 mg/ml, respectively), which were very close to those achieved by chlorhexidine (control). Apart from the *C. glabrata *clinical isolates, the average susceptibility to EOCM which was the lowest observed in this trial (MIC 3.75/MFC 7.5 mg/ml), isolates of *C. albicans *also demonstrated lower susceptibility, not only to EOCM (MIC 2.70/MIC 5.32 mg/ml) but also to EOBC_2_ (MIC 2.68/MFC 5.27 mg/ ml). However, the average susceptibilities of *C. krusei*, ATCC750 and ATCC10231 to EOCM, EOCLand EOBC_1_, as well of *C. albicans* to EOCL and *C. glabrata* to EOCL and EOCM, were all identical (MIC 2.5/MFC 5.0 mg/ml) (Table 2(Tab. 2)).

### Antifungal efficacy of essential oils

Among all tested essential oils, generally EOBC_2_ proved to be the most efficacious against all fungi at the lowest concentration applied; 2.5 mg/ml of this oil successfully killed 100 % of *C. krusei *(1 of 1 isolate), ATCC 750 (1 of 1 strain) and ATCC 10231 (1 of 1 strain), as well as 92.7 % of *C. albicans *(51 of 55 isolates), and 50 % of *C. glabrata *(1 of 2 isolates).

Similar trends in activity against *C. krusei*, ATCC 750 and ATCC 10231, were observed with EOCM, EOCL and EOBC_1_, but the concentrations required to achieve the same effect (100 % kill) was two-times greater (5 mg/ml). In addition, a concentration of 5 mg/ml in the case of EOCL and EOBC_1_ also achieved 100 % killing of *C. glabrata *isolates, but was not as efficacious in case of EOCM (50 % kill). 

Anti-candidal activity of the same essential oils against two *Boswellia *spp., was previously tested and confirmed (Abdoul-latif et al., 2012[1]), as well as a weak anti-candidal efficacy of EOCM (Carvalhinho et al., 2012[9]), in comparison to EOBC, although those oils, as well as many other oils and their major components were tested only against the ATCC 10231 strain (Hammer et al., 1999[16]; Tampieri et al., 2005[39]). Although this group did not describe the efficacy of oils against *Candida* spp. clinical isolates, their data were in accordance with our results with regard to the same ATCC strain. On the other hand, because of the small number of clinical isolates of *C. krusei* (1) and *C. glabrata* (2) recovered from human oral cavity, we are not in position to draw any serious conclusions on the efficacy of our essential oils, so our findings regarding *C. krusei* and *C. glabrata* isolates may serve only as a reference. In addition, our results also confirm that *C. albicans *was the most common oral cavity clinical isolate, while the isolates from *C. glabrata *and* C. krusei* were recovered not so frequently but consistently.

## Discussion

The main components of EOBC_1_ and EOBC_2 _were, typically dominant in monoterpenes (Abdoul-latif et al., 2012[1]). However, the two oils differed between themselves in their individual components percent contribution (Chiavari et al., 1991[10]; Abdulwahab et al., 1987[2]). Our EOCM differed from the literature in abundance of curzerene (Chiavari et al., 1991[10]), which was greater than the previously reported high percentage of furanoeudesma-1,3-diene (Baser et al., 2003[7]), although they were both present in similar and quite high percentages (34.65 and 32.77 %, respectively), as confirmed in other articles (Hanuš et al., 2008[17]; Baser et al., 2003[7]).

To the best of our knowledge, this study is the first to emphasize the susceptibility of several oral cavity clinical isolates and ATCC strains of *Candida* spp. to these four essential oils from the *Burseraceae* family. 

As out of all tested *Candida* spp. clinical isolates in our experiment, 5 to 10 % responded differently to applied treatment, including the control, we assumed that they undergone certain modifications, similarly to results of Zomorodian et al. (2011[45]) which reported 9.2 % of 206 *Candida* spp. isolates tested on diverse antifungal treatments showing minor mutations. This led us to conclusion that we should seriously take in consideration that it may be one of the reasons for antifungal treatment failure. 

In addition, the lower susceptibility of the clinical isolates of *C. albicans *in comparison to the ATCC 10231 strain to plant-derived essential oils, as shown in our research, is in accordance with findings from other studies (Abdoul-latif et al., 2012[1]), emphasizing, once again, the strength of *C. albicans *clinical isolates, also confirmed for isolates recovered from the throat and mouth (Paniagua et al., 2002[26]; Manfredi et al., 2006[24]). 

A successful therapy can be defined as “a suitable agent prescribed to treat the right organism at an appropriate dosage”. Epidemiological studies by surveillance to determine the true frequency of antifungal resistance may be the first step to control the emergence of antifungal resistance. Rapid identification of fungal pathogens and the measurement of the MIC of clinical isolates *in vitro *may be helpful. Knowledge gained from studying the mechanisms of antifungal resistance may provide ideas on how to limit the emergence of resistance to those marketed antifungal agents and to develop safer and better compounds for the next generation of antifungal agents (Anibal et al., 2010[5]). Therefore, new treatment strategies, especially natural, are urgently needed.

## Conclusion

In this paper, we encourage the development of new therapeutic agents against opportunistic fungi of the *Candida *genera, which, as do other microorganisms, have the ability to acquire resistance to antimicrobials, especially during prolonged treatments such as those associated with immunocompromised patients. 

Our results conclusively show susceptibility of *C. albicans*, *C. krusei* and *C. glabrata* oral cavity clinical isolates and ATCC 10231 and ATCC 750 strains to all tested commercial oils from the *Burseraceae* family. Although less efficacious on a mg/ml basis in comparison to the mouth rinse with chlohexidine 0.05 %, our findings suggest that the sample 2 of *Boswellia carterii *oil (EOBC_2_) could serve as an alternative, either alone or in combination with other antifungal agents, for preventive and/or therapeutic purposes, in patients prone to recurrent oral candidiasis.

In addition, our findings increase the knowledge about biological characteristics of *Candida* spp. isolated from the human oral cavity and we consider them very important with regard to their raising resistance to commercial antifungal drugs and consequent antifungal treatment failures. Therefore, this research should be understood as contributing to the overall endeavour of discovering alternative approaches to current therapeutic challenges.

## Acknowledgements

The authors appreciate financial support of the Ministry of Education, Science and Technological Development of Republic of Serbia (Grant № 173032).

## Conflict of interest

The authors declare that they have no conflict of interest.

## Figures and Tables

**Table 1:**
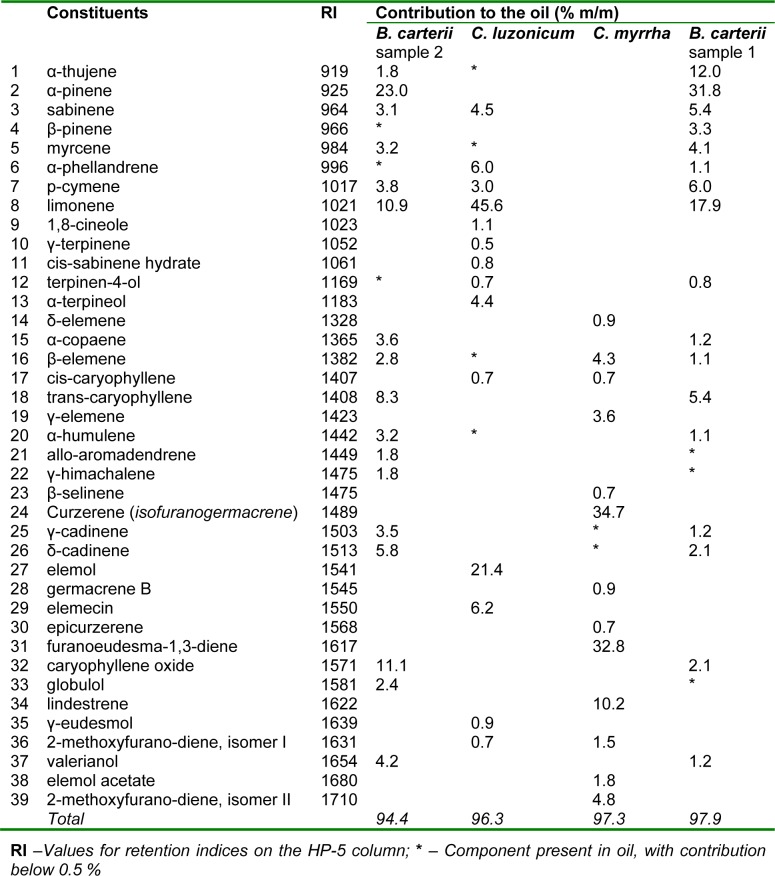
Chemical composition of four commercial oils from the *Burseraceae* family used in experiment (Presented components contribute to corresponding oils with 0.5 %).

**Table 2:**
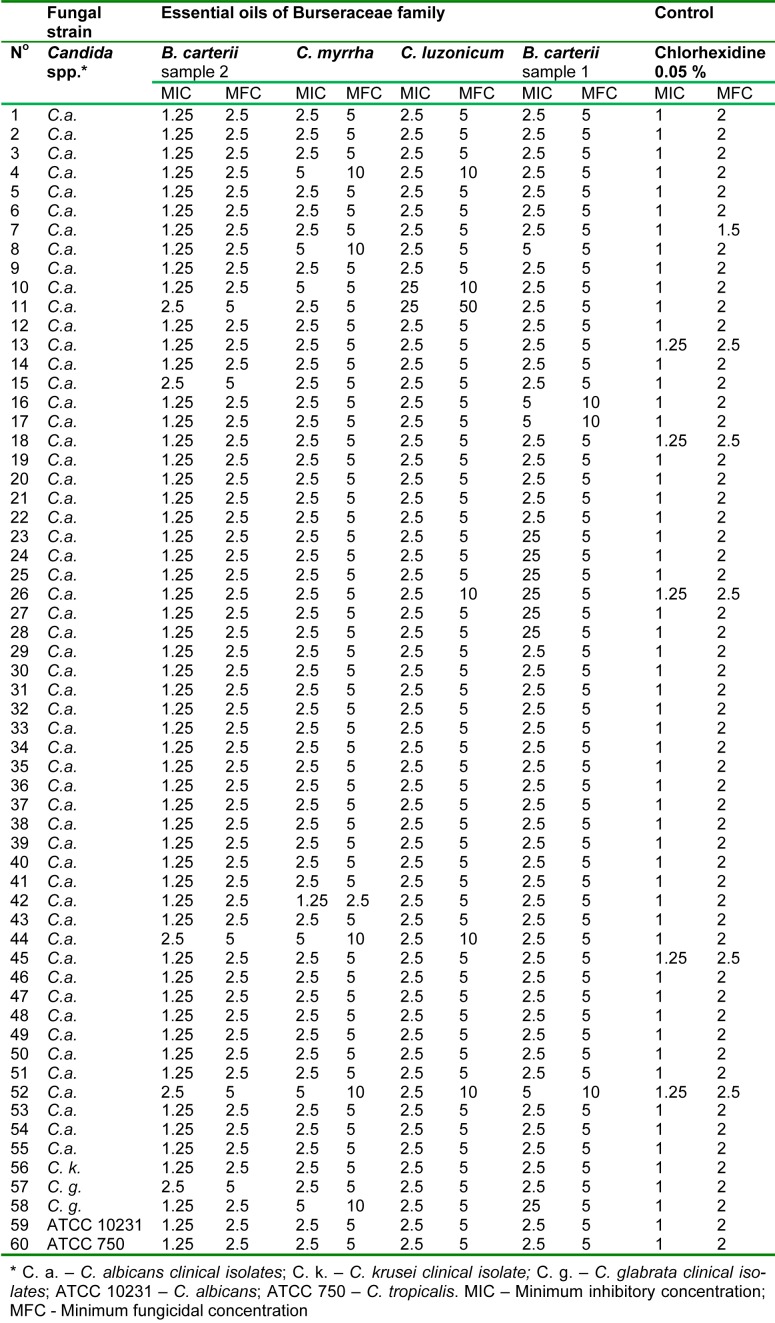
Anti-candidal activity of four essential oils from the *Burseraceae* family (mg/ml)
